# Hematopoietic stem cell metabolism within the bone marrow niche – insights and opportunities

**DOI:** 10.1002/bies.202400154

**Published:** 2024-11-06

**Authors:** Koen Kemna, Mirjam van der Burg, Arjan Lankester, Martin Giera

**Affiliations:** ^1^ Department of Pediatrics, Laboratory for Pediatric Immunology Willem‐Alexander Children's Hospital, Leiden University Medical Center Leiden The Netherlands; ^2^ Center for Proteomics and Metabolomics Leiden University Medical Center Leiden The Netherlands

**Keywords:** hematopoiesis, metabolism, stem cells

## Abstract

Hematopoiesis unfolds within the bone marrow niche where hematopoietic stem cells (HSCs) play a central role in continually replenishing blood cells. The hypoxic bone marrow environment imparts peculiar metabolic characteristics to hematopoietic processes. Here, we discuss the internal metabolism of HSCs and describe external influences exerted on HSC metabolism by the bone marrow niche environment. Importantly, we suggest that the metabolic environment and metabolic cues are intertwined with HSC cell fate, and are crucial for hematopoietic processes. Metabolic dysregulation within the bone marrow niche during acute stress, inflammation, and chronic inflammatory conditions can lead to reduced HSC vitality. Additionally, we raise questions regarding metabolic stresses imposed on HSCs during implementation of stem cell protocols such as allo‐SCT and gene therapy, and the potential ramifications. Enhancing our comprehension of metabolic influences on HSCs will expand our understanding of pathophysiology in the bone marrow and improve the application of stem cell therapies.

## Graphical abstract

HSC metabolism is tightly regulated by intrinsic processes and environmental factors within the bone marrow niche. Regulating the metabolic state of HSCs is crucial for preserving HSC fitness and upholding hematopoietic processes. Altered bone marrow niche environments, in states of disease, disrupt the metabolic state and fitness of HSCs.

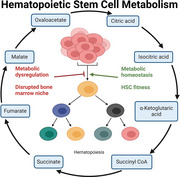

## INTRODUCTION

Hematopoiesis is the essential process sustaining the hematopoietic lineages, which unfolds within the bone marrow niche. At its core, Hematopoietic Stem Cells (HSCs) play a critical role, ensuring the long‐term repopulation capacity vital for continuous renewal of white blood cells, red blood cells, and platelets. The hypoxic environment of the bone marrow niche where HSCs reside is an important prerequisite in hematopoietic processes. Recently, cellular metabolism has taken center stage in this process, revealing profound influence on cell fate decisions and differentiation of HSCs. Next to that, the BM niche is upheld by many supporting non‐hematopoietic cell types and differentiating hematopoietic progenitors. All of these cells are subject to the intricacies of the BM niche including oxygen tension, nutrient availability, growth signals and cell to cell contact, ultimately driving hematopoiesis.

This review aims to shed light on the interconnectedness and importance of metabolism and hematopoiesis within the bone marrow niche. By emphasizing the intertwined roles of HSCs, cellular metabolism, and the hypoxic niche, we aim to provide a comprehensive perspective on the current state of knowledge.

## THE BONE MARROW NICHE AS METABOLIC ENVIRONMENT

**FIGURE 1:**
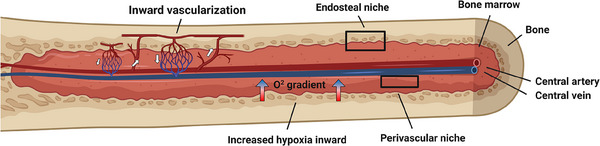
Representation of the vascular niches within the bone marrow.

Hematopoiesis takes place in the bone marrow (BM), found within the cavities of axial and long bones. Completely enclosed by bone, a unique environment essential for the upholding of blood cell formation is found, also named the BM niche. At the core of the BM niche lie the hematopoietic stem cells (HSCs), defined as multipotent primitive cells that can uphold all types of blood cells.^[^ ^1^ ^]^ The first experimentally defined concept for such a tissue stem cell came from Till and McCulloh in 1961–1964, who based much of their work on radiation of the mouse bone marrow and spleen colony‐forming assays in vivo.^[^ ^2^, ^3^, ^4^ ^]^ They demonstrated that individual cells have the ability to generate multiple lineages while maintaining the multipotent characteristics of the parent cell. Ray Schofield expanded on this in 1978, by first describing the stem cell niche hypothesis. Schofield recognized that spleen colony‐forming cells (CFU‐S) were less resilient than BM cells at reconstitution of hematopoiesis in animals, and suggested that stem cells needed to occupy their “niche” to function properly.^[^ ^5^ ^]^ Early studies proposed that bone‐lining cells, including osteoblasts and osteoclasts, occupying the endosteal regions predominantly shape BM niche for HSCs.^[^ ^6^, ^7^, ^8^ ^]^ More recently, it has been elucidated that vascularization plays a pivotal role in the BM, with vascular niches emerging as key regulators of HSCs. Preferentially in the endosteal regions, blood vessel capillaries proliferate and anastomose, forming a venous sinusoidal network that converges into the central vein.^[^ ^9^ ^]^ This vascular system forms specific BM vascular niches, with varying metabolic environments (Figure 1). As shown by Spencer et al. this niche is further subdivided into distinct metabolic regions, with the peri‐sinusoidal part exhibiting the greatest degree of hypoxia and the endosteal regions being comparatively less hypoxic.^[^ ^10^ ^]^ However, the BM is generally considered to be a hypoxic environment with low oxygen tension despite its high vascularity, a characteristic shared with metabolically active tumors. This illustrates a highly cellular and metabolically demanding environment in which hematopoiesis takes place.

Within this heterogeneous environment, HSCs evidently reside in perivascular compartments. Importantly, their maintenance is supported by secretion of factors by both endothelial cells and perivascular stromal cells.^[^ ^11^, ^12^ ^]^ The precise localization of HSCs among the vascular niches has been disputed, however. Some studies claim that dormant HSCs associated with small arterioles are preferentially found in the endosteal regions.^[^ ^9^, ^13^ ^]^ Others reported that non‐dividing HSCs were located in the vicinity of sinusoidal blood vessels, in the more hypoxic perisinusoidal niche.^[^ ^14^ ^]^ These disparate placements of HSCs could potentially be reconciled by propositions that localization is HSC subtype dependent. Indeed, several studies indicate that lymphoid‐biased HSCs appeared to locate near arterioles, while myeloid‐biased HSCs associate with megakaryocytes in the vicinity of sinusoids.^[^ ^15^, ^16^ ^]^ In general, these studies demonstrate the challenge of spatially localizing HSCs and argue for HSC subtype specific regions.

## METABOLISM OF HSCS IN THE BM NICHE

HSCs are commonly thought to play a crucial role in maintaining hematopoiesis, particularly in response to immune‐hematological challenges.^[^ ^17^, ^18^ ^]^ Continuous maintenance of the hematopoietic system and its functions ultimately relies on the “stemness” or stem cell potential of HSCs. Rossi et al. described the properties of this stemness, which include: self‐renewal potential, multi‐lineage differentiation potential and quiescence.^[^ ^19^ ^]^ The pool of HSCs can be further divided into long‐term self‐renewing HSCs (LT‐HSCs), temporarily self‐renewing short‐term HSCs (ST‐HSCs) and multipotent progenitors (MPP) that have lost the ability to self‐renew.^[^ ^20^, ^21^ ^]^ Of these HSC subtypes, it is LT‐HSCs that serve as the cornerstone of hematopoiesis, possessing the capacity for indefinite self‐renewal and ensuring the consistent production and renewal of blood cells.^[^ ^22^ ^]^ To maintain these functions, cellular metabolism is increasingly being recognized as fundamental characteristic and key regulator in HSC maintenance.^[^ ^23^, ^24^ ^]^ Further, we will primarily discuss LT‐HSCs.

### Dormant protection: Maintenance of quiescence in HSCs

In order to preserve their capacity for self‐renewal, HSCs must be protected from proliferative and genotoxic stress. Consequently, HSCs often exist in a state of quiescence.^[^ ^23^, ^25^, ^26^ ^]^ In the hypoxic microenvironment of the BM, HSCs rely on anaerobic glycolysis while limiting the tricarboxylic acid (TCA) cycle and mitochondrial oxidative phosphorylation (OXPHOS).^[^ ^27^, ^28^ ^]^ While HSCs contain higher numbers of mitochondria compared to lineage‐committed progenitors and mature hematopoietic cells, it is imperative for HSCs to control mitochondrial activity.^[^ ^29^, ^30^ ^]^ The main issue associated with mitochondrial OXPHOS lies in the generation of reactive oxygen species (ROS) as byproducts. Elevated ROS contributes to cytotoxicity by producing reactive free radicals,^[^ ^31^, ^32^ ^]^ regulating protein function through oxidization^[^ ^33^ ^]^ and modulating protein kinases and transcription factors.^[^ ^32^, ^34^ ^]^ Extensive evidence indicates that enhanced mitochondrial respiration and increased ROS levels reduce the self‐renewal capacity of LT‐HSCs, impairing their maintenance and functionality.^[^ ^27^, ^28^, ^35^, ^36^ ^]^

**FIGURE 2:**
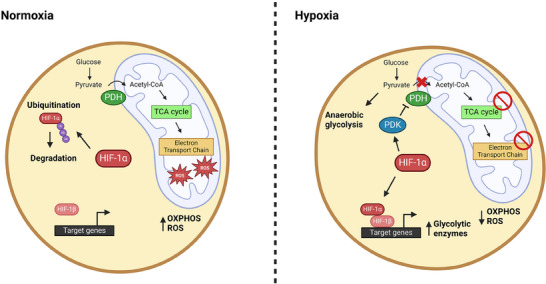
Overview of the metabolic changes induced by HIF‐1α under hypoxic conditions.

The metabolic switch towards anaerobic glycolysis is mediated in great part by hypoxia inducible factor (HIF)‐1 (Figure 2). Transcriptionally regulated by *Meis homeobox 1 (Meis1)*,^[^ ^28^ ^]^ HIF‐1 is highly elevated in LT‐HSCs and involved in the response and metabolic adaptation to hypoxic environments.^[^ ^37^, ^38^ ^]^ HIF‐1 activity relies on the dimerization of the constitutively active HIF‐1β and the tightly regulated HIF‐1α. Active HIF‐1 upregulates crucial glycolytic enzymes to promote anaerobic glycolysis. Rate‐limiting catalysts of glycolysis like Pfk‐1 were upregulated in HSCs.^[^ ^27^ ^]^ Notably, there was a reduction in the levels of glycolytic enzymes, leading to diminished glycolytic capabilities in HIF‐1α deficient cells.^[^ ^39^, ^40^ ^]^ Additionally, HIF‐1 limits mitochondrial OXPHOS through active suppression of the TCA cycle. In normoxia, pyruvate dehydrogenase (PDH) converts pyruvate into acetyl‐CoA which enters the TCA cycle and contributes to the respiratory chain. Hypoxic activation of HIF‐1 *trans*‐activates the genes encoding the pyruvate dehydrogenase kinase isoforms (PDK1‐4), which inactivate PDH and effectively disconnects glycolysis from the TCA cycle. Thereby HIF‐1 directs all glucose towards anaerobic glycolysis, limiting mitochondrial oxygen consumption and ROS production.^[^ ^27^, ^38^, ^41^, ^42^ ^]^ It is imperative to consider the environmental conditions on the regulatory role of HIF‐1. During normoxia, the HIF‐1a subunit is ubiquitinated and degraded.^[^ ^43^, ^44^ ^]^ While one study depicted a shift towards mitochondrial respiration and increased ROS production upon deletion of *Meis1*,^[^ ^45^ ^]^ two others demonstrated that the depletion of HIF did not ultimately affect HSC maintenance.^[^ ^46^, ^47^ ^]^ How essential HIF is in the retention of quiescence, and whether other factors are perhaps involved in the adaptation to hypoxic environments remains unclear. Nevertheless, exposure of HSCs to atmospheric oxygen was shown to increase ROS production and deplete HSCs.^[^ ^36^ ^]^ A recent study revealed shifts in cell metabolism, redox status and lipid composition of adenocarcinoma tumor cells. This highlights the extent to which oxygen availability can induce profound metabolic changes, some of which may be partly irreversible. Similar effects could be anticipated as HSCs respond to the oxygen availability.^[^ ^48^ ^]^

Together, these findings indicate that HSCs may not merely be contending with hypoxia, but that the hypoxic environment within the vascular niches could play a pivotal role in preserving their quiescence. However, a lack of conclusive evidence warrants further investigation into the regulation and influence of hypoxia on HSC fitness.

### Regulating mitochondrial dynamics is crucial for the maintenance of quiescence

Next to hypoxia, other intrinsic mechanisms implicated with HSC maintenance are autophagy, mitochondrial membrane potential and the unfolded protein response. Autophagy entails a cellular recycling mechanism which governs cellular health by eliminating damaged or abundant organelles and excess metabolites.^[^ ^49^ ^]^ Mitophagy, the specific removal of mitochondria, is especially important in reducing mitochondrial mass and respiration.^[^ ^50^ ^]^ As key orchestrators of mitophagy, Pten‐induced putative kinase 1 (Pink1) interacts with the E3 ubiquitin ligase Parkin (Park2) to directly facilitate mitochondrial removal via autophagosomes.^[^ ^51^ ^]^ By accumulating only on the surface of defective mitochondria, Pink1 and Parkin specifically sense and remove damaged mitochondria.^[^ ^52^, ^53^ ^]^ Disruptions in autophagic processes and dysregulation of key autophagy‐related genes have been linked to mitochondrial metabolism accompanied by HSC exhaustion and loss of repopulation potential.^[^ ^49^, ^54^ ^]^ Next to the regulation of mitochondrial abundance, it was also shown that regulating mitochondrial properties is pivotal in HSCs. By controlling the electron transport chain (ETC) elements, particularly with increased expression of ETC complex II, HSCs maintain high mitochondrial membrane potential and limit ATP generation through the aerobic TCA cycle. Deregulations of the ETC complex II resulted in lower mitochondrial membrane potential and accumulation of ROS, accompanied by diminished HSC fitness.^[^ ^55^, ^56^ ^]^ The unfolded protein response (UPR) entails a regulating response to unfolded and misfolded proteins. Accumulation of these proteins, caused by increased protein synthesis, triggers ER stress and necessitates the UPR managing cell death or survival.^[^ ^57^, ^58^ ^]^ The mitochondrial response, UPRmt, is implicated with cellular senescence and was activated when HSCs transitioned from their quiescent state.^[^ ^59^, ^60^ ^]^ Along these lines, Sirtuin 7 (Sirt7) was shown to suppress mitochondrial metabolism and govern HSC aging.^[^ ^61^ ^]^ Together, these mechanisms underline the importance of mitochondrial health to HSC functionality.

So far, we have argued that HSCs maintain quiescence by mainly depending on anaerobic glycolysis and limiting OXHPOS and ROS production. Nevertheless, some mitochondrial activity is upheld and appears indispensable. Two studies indicated that the disruption of mitochondrial respiration was also detrimental to HSC survival and maintenance.^[^ ^62^, ^63^ ^]^ Since glycolysis and the TCA cycle are mostly disconnected in HSCs, alternative nutrient pathways, specifically fatty acid and amino acid metabolism, may be of importance in facilitating the TCA cycle.^[^ ^64^ ^]^ Catabolism of branched chain amino acids and glutamine metabolism results in the production of α‐ketoglutarate, oxaloacetate, acetyl‐CoA and succinyl‐CoA, all of which can fuel the TCA cycle through subsequent steps.^[^ ^65^ ^]^ Next to glutamine, valine was shown to be essential for HSC homeostasis and maintenance in culture.^[^ ^66^ ^]^ The availability of amino acids can serve as limiting factor, controlling HSC activation and ensuring quiescence, as was shown for aspartate.^[^ ^67^ ^]^ Moreover, amino acid catabolism and a tight regulation of the protein synthesis rate were demonstrated to be crucial in the regulating of cellular proteostasis and the levels of amino acids, thereby maintaining HSC fitness and regenerative activity.^[^ ^68^, ^69^, ^70^ ^]^

Lipid metabolism, primarily fatty acid oxidation (FAO), also participates in HSC self‐renewal.^[^ ^71^, ^72^ ^]^ In addition, lipid metabolism plays a role in mitochondrial homeostasis. PPAR directed FAO pathways enhance the recruitment of Parkin in mitochondria, promoting mitophagy and maintaining mitochondrial quality.^[^ ^51^ ^]^ Consequently, the impairment of fatty acid transport and oxidation through deletion of PPAR‐delta resulted in diminished HSC repopulation potential.^[^ ^51^, ^71^ ^]^ These findings highlight the relevance of alternative metabolic pathways and metabolites in HSC quiescence and illustrate their involvement in the regulation of HSC proliferation and determination of cell fate.

### The distinction between asymmetric and symmetric division regulate cell fate

As HSCs transition from their dormant state and re‐enter the cell cycle, the daughter cell's fate is governed by the choice between symmetric and asymmetric division. Symmetric division yields two daughter cells with similar fate, leading to both cells returning to dormancy and expanding the pool of quiescent LT‐HSCs. States of stress and emergency hematopoiesis may bring forth two daughter cells of differentiating progeny, contributing to hematopoietic differentiation thus depleting the LT‐HSC pool. Alternatively, asymmetric division can occur where only one daughter cell undergoes differentiation and the other daughter cell returns to a dormant state.^[^ ^73^, ^74^ ^]^ It has been suggested that asymmetric division of organelles and lysosomes plays a role in maintaining a healthy HSC pool. Daughter cells receiving more lysosomes, crucial for autophagic processes, re‐enter a state of dormancy.^[^ ^75^ ^]^ Daughter cells inheriting dysfunctional or surplus mitochondria are coerced into cell cycle entry and differentiation, thereby alleviating the quiescent cell population of potential cellular damage.^[^ ^76^, ^77^ ^]^

At the same time, the asymmetric distribution of organelles may serve as important mitochondrial influence in determining cell fate and directing a daughter cell toward differentiation.^[^ ^78^ ^]^ Another factor of crucial importance in HSC division patterns is the PPAR‐FAO pathway. The deletion or pharmacological inhibition of PPAR‐δ and FAO resulted in symmetric division of HSC daughter cells. Moreover, the promyelocytic leukemia (PML) tumor suppressor protein was found to be a key regulator of PPAR‐δ directed FAO and is associated with HSC maintenance,^[^ ^71^, ^79^ ^]^ while PPAR‐δ activation promoted asymmetric cell division. The PML‐PPAR‐FAO pathway might therefore be an important metabolic switch controlling cell fate. This was underlined by the finding that CD36‐mediated fatty acid uptake is required for rapid HSC expansion under inflammatory stress. Without CD36‐mediated fatty acid uptake and consequent FAO, cells were unable to enter the cell cycle.^[^ ^80^ ^]^ Others observed that PML and FAO could maintain ATP levels in breast epithelial cells that detached from the extracellular matrix.^[^ ^81^, ^82^ ^]^ Based on this, Ito et al. proposed that the PML‐PPAR‐FAO pathway facilitates asymmetric division by ensuring an adequate ATP supply when HSCs lose contact with the niche during division.^[^ ^71^ ^]^ Studies of the transcriptome revealed the involvement of differential expression,^[^ ^83^ ^]^ alternative splicing^[^ ^84^ ^]^ and long non‐coding RNAs.^[^ ^85^ ^]^ Interestingly, one study indicated the enrichment of genes involved in cell cycle and metabolic growth even in quiescent HSCs. The authors hypothesized that the expression of these genes is upheld so these cells are primed for rapid activation when needed, despite their dormant state.^[^ ^83^ ^]^ If this is true, it further emphasizes the need for metabolic and cell cycle control. While these studies reveal interesting targets, like the metabolic enzyme Gls ^[^ ^86^ ^]^ or transcription factors GATA1^[^ ^87^ ^]^ and Meis1,^[^ ^83^, ^84^, ^85^ ^]^ conclusive evidence on the involvement of these processes remains elusive. Further investigation into the intricacies of asymmetric cell division are warranted. However, it appears evident that some form of asymmetric cell division is imperative for changing the fate of a daughter cell towards that of a differentiating progenitor.^[^ ^88^ ^]^

### HSC differentiation is facilitated by an aerobic switch

The transition of HSCs to a more proliferative state introduces new metabolic demands. Proliferating cells need to generate energy, facilitate the uptake of nutrients (e.g., lipids, amino acids, and nucleotides) and activate biosynthetic processes required for cell replication.^[^ ^89^ ^]^ The increased energy demand is met by a switch towards mitochondrial respiration and OXPHOS, a more efficient mechanism of ATP production. Cell cycle entry is associated with increases in mitochondrial biogenesis and maturation.^[^ ^90^ ^]^ Mitochondria undergo morphological changes to become more elongated and organize into an interconnected network, supporting energy exchange and nutrient supply.^[^ ^91^ ^]^ These changes coincide with shifts in fatty acid and amino acid metabolism, as the cellular metabolism machinery is reprogrammed to support an oxidative switch and differentiation.^[^ ^92^, ^93^ ^]^ The importance of transitioning towards OXPHOS for HSCs to facilitate differentiation was emphasized in a study focusing on protein tyrosine phosphatase mitochondrial 1 (PTPMT1).^[^ ^90^ ^]^ Inactivation of protein tyrosine phosphatase mitochondrial 1 (PTPMT1), a protein crucial for mitochondrial OXPHOS, resulted in a differentiation block and ∼40‐fold increase of the HSC pool, emphasizing the importance of an aerobic transition. As the energy demand increases, so does the need for nutrients and mitochondria. The PI3K/Akt/mTOR pathway, involved in nutrient sensing, mitochondrial biogenesis, and overall cell proliferation,^[^ ^94^ ^]^ was demonstrated to be of importance for the active cycling of HSCs.^[^ ^95^ ^]^ Constitutively active signalling of this pathway accelerated both the proliferation and depletion of HSCs.^[^ ^96^, ^97^ ^]^ In contrast, dysregulation of this pathway resulted in a differentiation block where HSCs remained in a quiescent state.

However, increased OXPHOS comes at the cost of increased ROS production. At high concentrations, ROS contribute to HSC aging and induce cell death.^[^ ^98^ ^]^ Nevertheless, controlled elevation of ROS is necessary to stimulate HSC proliferation and support hematopoiesis.^[^ ^99^, ^100^ ^]^ Studies looking into NAPDH oxidases (NOXs), a family of enzymes that actively produce ROS, elucidated an important role for redox signalling.^[^ ^101^, ^102^ ^]^ NOX‐related ROS are reportedly involved in cell cycle progression,^[^ ^103^ ^]^ mobilization,^[^ ^104^ ^]^ and differentiation^[^ ^105^ ^]^ of stem and progenitor cells. Together, these observations suggest that ROS function not merely as byproduct of OXPHOS, but serve as second messenger and govern cell fate. One way in which ROS achieve this is by HSC‐specific phosphorylation and activation of p38 MAPK ^[^ ^106^ ^]^ or the initiation of NF‐kB signalling through removal of IkB. Again, this signalling pathway is implicated in proliferation and apoptosis.^[^ ^107^ ^]^ Other targets undermined by ROS oxidization include the ATM‐FOXO dependent autophagy pathways,^[^ ^108^ ^]^ p53 dependent gene expression ^[^ ^109^ ^]^ and the BM‐1 polycomb protein.^[^ ^110^ ^]^ These pathways have in common that they regulate the generation of ROS in redox homeostasis. Consequently, elevation of ROS levels is suggested to occur through positive feedback mechanisms, where increased ROS levels induce additional ROS release.^[^ ^101^ ^]^ In general, the transition to aerobic mitochondrial activity places HSCs into a heightened oxidative state which drives differentiation. Recent studies have implicated 7‐DHC, a cholesterol precursor, to play a crucial role in the cellular protection against ROS.^[^ ^111^, ^112^ ^]^ 7‐DHC functions by absorbing excess ROS, thereby protecting lipids from oxidative damage and mitigating ROS‐induced ferroptosis. These findings illustrate a novel intrinsic mechanism that counters oxidative damage through ROS interception, prompting further investigation into the potential involvement of similar protective mechanisms in HSCs.

In summary, it has become evident that metabolic regulation, including the shift towards anaerobic glycolysis, mitochondrial control and ROS homeostasis, is crucial in preserving HSC quiescence and repopulation potential. The initiation of HSC differentiation occurs through asymmetric division, thereby governing cell fate. Differentiation processes are facilitated by a transition towards aerobic metabolism, which is accompanied by increased nutrient uptake, energy production, and ROS signalling. Whether in quiescent or differentiative states, metabolic control is governed by intrinsic mechanisms and integral to the functioning of HSCs.

## MICROENVIRONMENTAL INFLUENCES OF THE BM NICHE ON HSC METABOLISM

In the previous chapter we discussed the metabolic processes of HSCs during quiescence and differentiation, with particular emphasis on the intrinsic mechanisms governing HSC metabolism and fate. However, HSCs do not exist in isolation. Rather, they reside within a more complex microenvironment where various cell types and factors coexist and interact. In this chapter, we will describe key external influences of the microenvironment on HSC metabolism (Table 1).

### Endothelial barrier and interactions

Endothelial cells (ECs) form the inner lining of blood vessels. Given that large numbers of HSCs are located in close proximity to the BM vasculature, an important role is ascribed to ECs in the BM niche.^[^ ^9^, ^14^, ^113^, ^114^ ^]^ We have previously discussed the importance of vascularization in creating distinct vascular niches suitable for hematopoiesis. The endothelial barrier also serves as a conduit for cells and metabolites. The passage of oxygen and ROS seems particularly significant in ECs, with increased ROS production being linked to vascular leakiness.^[^ ^115^ ^]^ ECs themselves rely predominantly on glycolysis instead of OXPHOS, further regulating ROS production. Consequently, the endothelial barrier plays an important role in modulating the metabolic composition of the BM, especially concerning ROS levels.^[^ ^116^ ^]^

Some studies suggest that ECs serve different roles depending on their location, arteriolar (AECs) or sinusoidal (SECs). For instance, it was shown that AECs were responsible for the secretion of all detectable stem cell factor (SCF), but not SECs.^[^ ^117^ ^]^ In turn, SECs demonstrated increased production of CXCL12 and adhesion molecules.^[^ ^118^ ^]^ Lastly, Itkin et al. demonstrated that arteriolar regions contained lower ROS levels, while higher ROS levels could be found near sinusoids.^[^ ^119^ ^]^ Together, this proposes a model in which the arteriolar and sinusoidal niches assume distinct roles in HSC cycling. Dormant HSCs reside in the endosteal regions near arterioles, while sinusoidal regions in the perivascular niche promote cycling and trafficking of HSCs to and from the bone marrow.^[^ ^119^, ^120^, ^121^ ^]^ While it has been accepted that ECs are vital supporters of HSCs in the BM niche, no direct link between EC metabolism and potential metabolic cross‐talk with HSCs has been established to the best of our knowledge.

### Mesenchymal stromal cells, a range of influence

Mesenchymal stromal cells (MSCs) are a group of multipotent cells implicated mostly with support and repair of connective tissues.^[^ ^122^ ^]^ MSCs are of great importance in forming the BM niche with their ability to differentiate into osteocytes, adipocytes and chondrocytes, as well as their supportive and immunomodulatory functions.^[^ ^123^ ^]^ The population of mesenchymal cells, termed CXCL12‐abundant reticular (CAR) cells,^[^ ^124^, ^125^ ^]^ which overlap with leptin receptor‐expressing (Lepr+) cells,^[^ ^11^ ^]^ function as mesenchymal stem cells (MSCs) and are a major component of the HSC niche.^[^ ^126^ ^]^ CAR/LepR+ cells express high levels of transcription factors Foxc1 and Ebf3, which are essential for maintaining the MSC population within the HSC niche by inhibiting adipogenic differentiation and preserving self‐renewal.^[^ ^127^, ^128^ ^]^ Additionally, some studies suggest that a rare population of nestin‐GFP expressing cells are MSCs forming HSC niches.^[^ ^129^ ^]^ However, other research disputes this, arguing that nestin is expressed in endothelial cells, not CAR/LepR+ cells, in both mice and humans.^[^ ^126^, ^130^, ^131^ ^]^ Within the BM, these cells exist in limited numbers and are positioned perivascularly, adjacent to the surfaces of blood vessels.^[^ ^9^, ^129^, ^132^ ^]^ Their primary contribution to HSCs lies in the expression of SCF and CXCL12, and depletion of these factors results in HSC depletion.^[^ ^9^, ^11^, ^12^, ^133^ ^]^ Additionally, MSCs support the BM niche with the production of pro‐angiogenic factors, growth factors, immunomodulatory cytokines, and extracellular vesicles.^[^ ^134^, ^135^ ^]^

Cellular metabolism has been linked to the functioning of MSCs, especially with regards to their immunomodulatory properties. Multiple studies have provided evidence supporting the involvement of the kynurenine pathway (KP) and indoleamine 2,3‐dioxygenase (IDO), key components of tryptophan metabolism, in the immunosuppressive effects exerted by MSCs.^[^ ^136^, ^137^ ^]^ Examples of other metabolic pathways implicated with MSC immunomodulation are the HIF‐1α mediated response to hypoxia, nitric oxide (NO) metabolism involving heme‐oxygenase‐1 (HO‐1) and NOS, and PPAR regulated lipid and glucose homeostasis including switches to aerobic metabolism.^[^ ^137^, ^138^ ^]^ Next to this, metabolic distress and the accumulation of ROS levels was shown to impair stromal niche function and decrease the support for HSCs in vitro and in vivo. Moreover, it was shown that MSCs can rescue lymphoblastic leukemia cells from oxidative stress and ROS by mitochondrial transfer.^[^ ^139^ ^]^ It is plausible that similar mechanisms occur in the bone marrow, where MSCs would protect HSCs from oxidative stress, thereby further integrating the metabolic regulation and fate determination of MSCs and HSCs.^[^ ^140^ ^]^

Another metabolically relevant characteristic of MSCs is their ability to produce colony stimulating factors (M‐CSF, GM‐CSF, G‐CSF).^[^ ^141^ ^]^ These are potent chemotactic factors affecting HSCs and MSCs. Phenotypic effects of these factor includes for instance the mobilization of MSCs after trauma ^[^ ^142^ ^]^ or the mobilization of HSCs and facilitating their entry into the circulation.^[^ ^143^ ^]^ Next to the phenotypic changes exerted by colony stimulating factors they have been linked to significant metabolic rewiring, initially described in macrophage subpopulations arising from either M‐CSF or GM‐CSF treatment.^[^ ^144^ ^]^ The connection between colony stimulating factors, metabolism and cell fate decisions raises the question in how far metabolic intricacies are reflective or potentially causative for specific cell trajectories. To this end, more research is required to shed light on the exact relation between MSC metabolism and HSC status.

### Osteoblasts metabolism is largely unmapped

Osteoblasts are part of the bone lining cells, located in the endosteum covering bone surfaces. These cells play a vital role in both bone resorption and formation, essential for overall bone health. In contrast to osteoclasts, which participate in bone resorption, osteoblasts contribute to the synthesis and secretion of extracellular proteins constituting the bone matrix.^[^ ^145^, ^146^ ^]^ Early investigations into HSCs and the BM niche pointed towards an endosteal niche in which osteoblasts were an important population directly connected to HSC maintenance. The main suggestions were that osteoblasts retained and supported dormant HSCs in the endosteal niche with the production of N‐cadherin, CXCL12, bone morphogenic protein (BMP), and Notch ligands.^[^ ^6^, ^147^ ^]^

However, recent studies failed to detect N‐cadherin expression in HSCs ^[^ ^148^, ^149^ ^]^ and conditional deletion of N‐cadherin from osteoblasts had no effect on HSC frequency.^[^ ^150^ ^]^ This undermined the role of N‐cadherin, previously thought to be an important adhesion molecule for osteoblasts. Deletion of CXCL12 and SCF from osteoblasts did not affect the HSC population.^[^ ^11^, ^151^ ^]^ Kunisaki et al.^[^ ^9^ ^]^ and Asada et al.^[^ ^152^ ^]^ added to this by demonstrating it was the stromal cells and not the osteoblasts who were responsible for the production of CXCL12.

Instead, research focus has shifted towards indirect support provided by osteoblasts in the BM niche. A plethora of studies illustrate the role of osteoblasts in signalling with the secretion of various molecules including Osteopontin (OPN),^[^ ^153^ ^]^ Thrombopoietin (TPO),^[^ ^154^ ^]^ Angpt‐1,^[^ ^155^ ^]^ and Wnt ligands.^[^ ^156^ ^]^ However, it was demonstrated that the deletion of Angpt‐1 and TPO in osteoblasts had no effect HSC maintenance, indicating that osteoblast signalling may not be essential.^[^ ^157^, ^158^ ^]^ Osteoblasts do play a pivotal role in shaping the BM niche, where their involvement in bone formation is critical for supporting vasculature and HSCs.^[^ ^159^, ^160^ ^]^ besides their structural role, however, the involvement of osteoblast metabolism and its potential metabolic influences exerted upon HSCs by osteoblasts remain largely unmapped.

### Adipocytes, roles yet to be defined

Adipose tissue in the bone marrow contributes to both the structural and regulatory aspects of the BM niche. In the BM, adipocytes are present in abundance and play an active role in metabolic regulation, secretion of factors and the structure and remodeling of the BM.^[^ ^161^ ^]^ The effect which adipocytes exert on the process of hematopoiesis is still under debate, as many findings seem contradictory or even paradoxical.

On one hand it has been suggested that BM adipocytes support HSCs. Studies showed that BM adipocytes promoted HSC survival and proliferation in vitro.^[^ ^162^ ^]^ Then, BM adipocytes were shown to produce SCF and promote hematopoietic regeneration after myeloablation.^[^ ^163^ ^]^ On top of that adipocytes also produce adiponectin,^[^ ^164^ ^]^ leptin,^[^ ^165^ ^]^ IL‐6 and IL‐8^[^ ^166^ ^]^ which are suggested to support hematopoiesis. In contrast, adipocytes were initially identified as negative regulators of hematopoiesis. Reduced numbers of HSCs were found in adipocyte rich BM compared to adipocyte free BM in mice.^[^ ^167^ ^]^ Additionally, hematopoietic regeneration was accelerated in fatless mice after transplantation,^[^ ^167^ ^]^ chemotherapy, ^[^ ^168^ ^]^ or irradiation.^[^ ^169^ ^]^ Lastly, adipocyte infiltration into the BM niche negatively impacts HSC function, and is characteristic of aged and metabolically dysfunctional BM.^[^ ^170^, ^171^ ^]^ This is accompanied by increased levels of free fatty acids, oxidative stress, and inflammatory responses, all of which are detrimental to HSC maintenance.^[^ ^172^, ^173^ ^]^

Possibly, some of these contradictory findings regarding adipocytes could be due to their role in regulating the lipid composition in the BM. BM adipocytes are metabolically active, illustrated by high glucose uptake. They are involved in the uptake and esterification of fatty acids, de novo lipogenesis and the regulation of FAO. In the BM, adipocytes exhibit reduced lipid metabolism and increased regulation of FAO and OXPHOS.^[^ ^174^, ^175^ ^]^ We have previously described the importance of lipid metabolism and PPAR‐FAO in HSC metabolism and fate decisions. Adipocytes could therefore serve a key role in modulating the BM niche and HSCs by conserving the balance of lipid metabolites. Disruptions of the lipid balance, either due to a deficit or excess of adipocytes, could result in contrasting effects that are equally harmful to the functioning of HSCs.

In summary, it has been widely accepted that BM adipocytes contribute to hematopoiesis through signalling.^[^ ^176^ ^]^ However, the extent to which adipocytes can regulate hematopoiesis through metabolic control is still unclear, especially when an excess of adipocytes is present as seen in obesity and ageing. Further research is required to untangle all the intricacies of BM adipocytes and their effect on HSC metabolism.

It should be noted that the bone marrow is a complex environment retaining many intricate populations and cell types. To discuss all of these factors is out of the scope of this review. Other external factors influencing the metabolism of HSCs in the BM niche include megakaryocytes,^[^ ^177^ ^]^ macrophages,^[^ ^178^ ^]^ neural regulation,^[^ ^179^ ^]^ the extracellular matrix (ECM),^[^ ^121^, ^180^ ^]^ and dietary nutrients and vitamins,^[^ ^181^ ^]^ which have been reported elsewhere.

## CHALLENGES AND FUTURE PERSPECTIVES IN HSC METABOLISM

### A complex metabolic environment: To what extent does metabolism determine cell fate?

Growing evidence suggests a significant contribution of cell metabolism to cell fate decisions, differentiation, and the regulation of effector functions. In this review, we discussed the extent to which metabolism regulates HSCs in the BM niche. However, a critical question remains: to what extent does cellular metabolism actually dictate the trajectory of cells? Or are metabolic changes simply a reflection of and induced by other dictating factors like epigenetics, cell‐cell signalling, and cytokines?

Much of the fate decisions in HSCs are governed by epigenetics. Chromatin accessibility and epigenetic modifications are involved in the regulation of differentiation commitment and cell cycle entry.^[^ ^78^ ^]^ This is further demonstrated by studies showing that chromatin remodeling occurs before the transcriptional differentiation process,^[^ ^182^ ^]^ and that lineage‐specific DNA methylation patterns can be observed.^[^ ^183^ ^]^ Moreover, these epigenetic changes are conserved in daughter cells after divisions via “mitotic memories.” Together, these studies illustrate the importance of epigenetics.^[^ ^74^ ^]^ However, we have also discussed some ways in which cell metabolism can alter cell fate. For instance, the PML‐PPAR‐FAO pathway and CD36‐mediated fatty acid uptake which regulate symmetry of division and cell cycle entry, respectively. Another example entails the mitochondrial control and ROS homeostasis which mediate anaerobic glycolysis and preserve HSC quiescence. Additionally, several studies have demonstrated that metabolism can alter and regulate epigenetics,^[^ ^184^, ^185^ ^]^ further illustrating the importance of metabolism.

The involvement of metabolic pathways in HSC fate decisions is evident, however cellular metabolism can be heavily influenced by the environment. HSC specific examples include the fatty acid availability as rate limiting factor in FAO, or the retention of HSCs in their hypoxic, quiescent niche for ROS homeostasis. Nutrient availability, nutrient sensing and external signalling are of great importance in decisions of cell fate. Recently, a review on T cell fate emphasized the importance of metabolic signalling for this process.^[^ ^186^ ^]^ The authors suggested a model in which nutrient sensing and direct signalling by metabolites created a microenvironment where cells become intertwined and exhibit social metabolic control. Perhaps it is the microenvironment which, through social control, shapes the metabolic and epigenetic identity of HSCs. In this model, HSCs would have to be well adapted to their environment to survive. Or from a more evolutionary perspective, unadjusted HSCs diminish while more adjusted HSCs flourish and populate the bone marrow.

These suggested models highlight the challenges in determining the involvement of metabolic microenvironments in the bone marrow. At the same time, they illustrate potential for therapeutic strategies. As we unravel the intricacies of metabolic influence in the bone marrow, our objective should be to improve the modulation of HSCs and their environment to optimize HSC integrity and functioning.

### Dysfunctional hematopoiesis: The role of inflammation in the BM

So far, we have described many intricacies of HSC metabolism in healthy bone marrow. However, in many patients suffering from hematological or bone marrow disorders, the bone marrow environment is characterized by a stressed or inflamed state. It is therefore crucial to unravel the impact of inflammatory events to the bone marrow and its effects on HSCs and hematopoiesis.

Inflammation can originate from bacteria, viral infections or damaged cells, resulting in pro‐inflammatory cytokines and immunogenic antigens. Alternatively, autoreactive self‐antigens can dysregulate the immune response and trigger auto‐immunity. Furthermore, imbalances in metabolism can induce excessive redox responses leading to ROS mediated immune responses. Inflammatory triggers and responses are complex and variable, the details of which have been reviewed elsewhere.^[^ ^187^, ^188^ ^]^ In general, inflammation causes an increased demand for short‐lived myeloid cells. Hematopoietic differentiation is skewed towards the myeloid lineages, at the expense of lymphoid progenitors. HSCs are subjected to inflammation‐enhanced proliferative stress, the accumulation of damage to DNA and organelles and the induced mobilization towards the bloodstream. These processes are accompanied by metabolic transitions from anaerobic glycolysis to mitochondrial respiration and OXPHOS, facilitating the active cycling of HSCs. The depicted response is largely triggered by inflammatory cytokines, particularly IFN‐γ, TNF‐α, and IL‐1β, making it a direct consequence of inflammation. In a chronically inflamed setting, there are baseline increases of these pro‐inflammatory cytokines. Prolonged inflammation continuously promotes proliferation, while the effects accumulate and impair long‐term self‐renewal in HSCs. It is this chronic exposure of HSCs to inflammatory stimuli that ultimately leads to functional decline. A recent article by Passegué et al. demonstrated that inflammation drives glycolytic impairment in aging HSCs. Interestingly, they show that autophagy functions as a cytoprotective mechanism by countering the glycolytic suppression during chronic inflammation, and that inducing autophagy improves HSC regenerative potential.^[^ ^189^ ^]^

We have summarized how the BM niche governs HSC metabolism and that this is intertwined with proliferation and mobilization. We have also stressed that limiting these processes and the accompanying metabolic transition is crucial for the long‐term self‐renewal and repopulation potential of HSCs. However, it is not only the hematopoietic progenitors that are subject to inflammatory stimuli. MSCs, for instance, are also pushed towards proliferation and mitochondrial respiration.^[^ ^187^ ^]^ Generation of ROS can even mediate mitochondrial transfer from MSCs to HSCs, facilitating the metabolic transition.^[^ ^190^ ^]^ As many non‐hematopoietic cells respond to inflammation, the entirety of the BM niche is remodeled to promote proliferation and differentiation at the cost of self‐renewal.

With that in mind, a clear picture emerges of how inflammation can disrupt the BM niche and deregulate hematopoiesis at its core. Moreover, one can envision the involvement of inflammation in numerous hematological disorders. Inflammatory triggers have been associated with conditions such as myelodysplastic syndromes,^[^ ^191^ ^]^ chronic granulomatous disease,^[^ ^192^ ^]^ ataxia telangiectasia,^[^ ^193^ ^]^ and aplastic anemia.^[^ ^194^ ^]^ Obesity has also been considered as an inflammatory disease that reshapes the BM.^[^ ^187^, ^195^ ^]^ Adipocytes are a source of inflammatory factors, while free fatty acids induce metabolic maladaptation and preferential differentiation of MSCs into adipocytes impairs osteogenesis. Collectively, these actions result in a less supportive BM niche for HSCs. The infiltration and accumulation of adipocytes in the BM impairs hematopoietic functioning. Interestingly, during the process of aging there is a gradual shift towards myeloid differentiation accompanied by a decline in lymphoid immune function.^[^ ^196^ ^]^ Also, it is well established that aging is linked to chronic low‐grade inflammation unrelated to infections, commonly referred to as “inflammaging.”^[^ ^195^ ^]^ Given the similarity of effects of aging and inflammation, a plausible inference arises that these phenomena are connected, with “inflammaging” emerging as a pivotal factor contributing to hematopoietic challenges during aging.

Moving forward, it is crucial to consider the role of inflammation in conditions where hematopoietic processes are affected. The significance lies not only in the direct impact of inflammation on hematopoietic lineages but also in its ability to metabolically and structurally reshape the BM niche, leading to altered hematopoiesis. Moreover, exploring therapeutic strategies that target the effects induced by inflammation, like the induction of autophagy, holds promise for addressing hematological and bone marrow disorders.

### Effective treatment: How do our treatment techniques influence HSC metabolism?

For many hematological and bone marrow diseases the treatment options include the use of HSCs from donor origin, also called allogeneic stem cell transplantation (allo‐SCT). Technological advancements have allowed us to mobilize, isolate and transfer healthy HSCs to patients with defects in certain hematopoietic processes, and successfully treating them. These techniques do require specific protocols in which abnormal conditions are forced upon the HSCs before they return to the BM niche. To benefit safe and successful treatment, it is therefore important to consider how these techniques might influence the characteristics of HSCs. Is the metabolism of HSCs affected by certain steps in the process of transplantation? What are the effects on long term HSC health and repopulation potential? And do we actually know what type of HSC we are administering to patients?

During allo‐SCT, donor HSCs are often harvested using G‐CSF therapy. This chemotactic factor induces proteolytic degradation of CXCL12 in the BM, thus decreasing the retention of HSCs in the niche and facilitating mobilization of HSCs into blood vessels.^[^ ^197^ ^]^ Here, we have discussed a model in which localization, metabolic transitions and differentiation are linked. According to the depicted model, quiescent HSCs are found primarily in the arteriolar niche. Their transition towards metabolically active, cycling HSCs coincides with localization towards the sinusoids and entry into the circulation. As G‐CSF therapy also induces this mobilization into the circulation, what can we expect from the induced metabolic response? Bernitz et al. investigated the effects of G‐CSF^[^ ^198^, ^199^ ^]^ and demonstrated that G‐CSF induced mobilization without stimulating proliferation in the CD41 negative population, representing the important LT‐HSCs. However, G‐CSF mobilized HSCs did have some competitive defects, as they exhibited a third of the repopulating potential of BM HSCs. It appears that mobilized HSCs may contain diminished functional potency, stemming from factors beyond proliferation. It remains unknown what metabolic changes are induced, and how HSC fate is altered. During the mobilization and harvesting of HSCs, these cells encounter distinct environmental conditions compared to the BM niche. Subsequently, HSCs are collected and maintained under normoxic conditions, or alternatively, subjected to (cryo)preservation and subsequent thawing before administration. It was shown that brief exposure to ambient air reduced the long‐term repopulation potential of HSCs, and mitigation of this oxygen shock could ameliorate this effect.^[^ ^36^ ^]^ It is therefore important to consider if HSCs retain their important quiescent state during these transitions. Can they ultimately revert to quiescence without accumulated damage? What impact can we expect on the engraftment and immune reconstitution and can we improve these processes? An intriguing example involves recent findings that the deletion or modulation of sphingolipids activates autophagy pathways and stabilizes HIF‐1a to regulate aerobic shifts, thus improving the metabolic fitness of HSCs.^[^ ^42^, ^200^ ^]^ Could therapeutic interventions targeting the metabolic fitness enhance the efficacy of HSC‐based therapies in the near future?

These questions are not only applicable to allo‐SCT, but could also be crucial in other techniques that target HSCs. Gene therapy, for example, or the in‐vitro expansion and engineering of HSCs are upcoming technologies. Moving forward with these therapies, the conditions forced on HSCs and the effects on metabolism and fate decision should be held in high regard. Besides that, we should aim to discover crucial and telling pathways that give indications on HSC metabolic health. Certain metabolites or other measures might find use as biomarkers, to serve as quality control for the efficacy and downstream effects of transplantation or gene therapy strategies. In general, a better understanding of HSC metabolism and fate decision will provide a broader base to adapt and improve treatment techniques. Our focus lays on clinical translation, increasing the success rates of current therapies and developing novel treatment options that may eventually be the standard of care in future stem cell therapy.

## AUTHOR CONTRIBUTIONS

This article was jointly conceptualized and written by all authors.

## CONFLICT OF INTEREST STATEMENT

The authors declare no competing interests.

**TABLE 1:** Overview of crucial pathways involved in HSC metabolism within the bone marrow.

Cell type	Effect	Pathway	Mechanism	References
Hematopoietic stem cells (HSCs)	Retains quiescence Limits cellular damage	Hypoxia induced HIF‐1a activation	Anaerobic glycolysis—limitation of mitochondrial OXPHOS and ROS generation	[27, 28] [37, 38, 39, 40, 41, 42, 43, 44, 45, 46, 47, 48]
		Autophagy and mitophagy	Removal of excess metabolites and damaged or abundant organelles	[49, 50, 51, 52, 53, 54]
		Unfolded protein response (UPR)	Regulation of unfolded and misfolded proteins	[57, 58, 59, 60]
	Regulates cell cycling	Asymmetric division	Asymmetric distribution of organelles, lysosomes and signaling molecules among quiescent and cycling daughter cells	[73, 74, 75, 76, 77, 78] [83, 84, 85, 86, 87, 88]
		PPAR—Fatty Acid Oxidation	Facilitates and promotes asymmetric division	[51, 71, 72] [79, 80, 81, 82]
	Induces proliferation and differentiation	Mitochondrial OXPHOS PI3K/Akt/mTOR pathway	Aerobic transition—increased nutrient uptake, energy production and biosynthetic processes facilitate proliferation	[89, 90, 91, 92, 93, 94, 95, 96, 97]
		ROS signaling NOX, p38 MAPK, NF‐κB	Increased ROS levels as byproduct of OXPHOS further induce proliferation as second messengers	[99, 100, 101, 102, 103, 104, 105, 106, 107, 108, 109, 110]
Endothelial cells	Regulates ROS generation	Endothelial barrier function	Endothelial cells regulate the passthrough of ROS, oxygen and metabolites	[115, 116]
	Maintenance of HSCs	Production of SCF and CXCL12	SCF and CXCL12 are crucial for the retention and continual support of HSCs	[117, 118]
Mesenchymal stromal cells (MSCs)	Maintenance of HSCs	Production of SCF and CXCL12	SCF and CXCL12 are crucial for the retention and continual support of HSCs	[9, 11, 12, 133]
	MSC immunomodulation	Tryptophan metabolism and the kynurenine pathway (KP)	The KP regulates immunomodulatory functions of MSCs and thus influences the BM niche environment	[134, 135, 136, 137, 138]
	Mobilization of HSCs	Production of CSF	CSF induces enzymatic degradation of CXCL12, thus diminishing their retention in the BM	[141, 142, 143, 144]
Osteoblasts	Supporting the BM niche	BM structure and supporting factors	Osteoblasts are crucial in structurally shaping the BM niche and secreting various supportive factors	[145, 146] [153, 154, 155, 156, 157, 158, 159, 160]
Adipocytes	Supporting hematopoiesis	Production of supportive factors	Adipocytes produce SCF, adiponectin, leptin, IL‐6 and IL‐8. These factors are all suggested to support hematopoiesis	[163, 164, 165, 166]
	Declining HSC maintenance	Excess adipocyte infiltration in the BM	Excess adipocytes are accompanied by increases in free fatty acids, oxidative stress and inflammation	[167, 168, 169, 170, 171, 172, 173]
	Maintaining lipid balance	Lipid uptake and regulation	Adipocytes take up fatty acids and regulate de novo lipogenesis and FAO. All of which are important for regulating the lipid composition	[174, 175]

## Data Availability

Data sharing not applicable to this article as no datasets were generated or analyzed during the current study
